# Dual Trigger Compared with Human Chorionic Gonadotropin Alone and Effects on Clinical Outcome of Intracytoplasmic Sperm Injection 

**DOI:** 10.22074/IJFS.2021.135720.1010

**Published:** 2021-10-16

**Authors:** Bahar Shakerian, Engin Turkgeldi, Sebile Guler Cekic, Sule Yildiz, Ipek Keles, Baris Ata

**Affiliations:** 1 Department of Obstetrics and Gynaecology, Koc University Hospital, Istanbul, Turkey; 2 Isfahan Fertility and Infertility Centre, Isfahan, Iran; 3 Department of Obstetrics and Gynaecology, Koc University, Faculty of Medicine, Istanbul, Turkey

**Keywords:** Dual Trigger, GnRH Agonist, Human Chorionic Gonadotropin, Infertility, *In vitro* Fertilisation Outcome

## Abstract

**Background:**

This study compared outcomes of the standard 6000 IU human chorionic gonadotropin (hCG) trigger with a dual trigger comprised of 6000 IU hCG and 1 mg leuprolide acetate for final oocyte maturation in an intracyto- plasmic sperm injection (ICSI) cycle. By convention, ICSI was performed in most cases at the clinic.

**Materials and Methods:**

In this retrospective study, a total of 50 women were included in each arm. Participants were matched for age, indication and number of prior assisted reproduction technology (ART) cycles. Women at risk for ovarian hyperstimulation syndrome (OHSS) were excluded. A flexible gonadotropin releasing hormone (GnRH) antagonist protocol was used and final oocyte maturation was triggered when two leading follicles were >17 mm. Distribution of variables was evaluated visually with histograms. Continuous variables were defined by mean (stand- ard deviation) or median (25^th^-75^th^ percentile) depending on distribution characteristics. Categorical variables were defined by numbers and percentages. Continuous variables were compared between the groups with the t test or Mann-Whitney U test as appropriate. Categorical variables were compared by the chi-square test and its derivatives as appropriate. A two-sided P<0.05 indicated statistical significance.

**Results:**

Both groups had similar antral follicle counts, median parity (0) and number of previous failed cycles (0). The median number of oocytes (8 vs. 7), metaphase-two oocytes (6 vs. 5.5), blastocysts (1 vs. 1), clinical pregnancy rates (CPR) (28% vs. 22%), ongoing pregnancy rates (OPR) (22% vs. 20%) and pregnancy rate per transfer (53.3% vs 53.8%) were similar between the dual trigger and hCG only groups, respectively.

**Conclusion:**

Dual trigger for oocyte maturation stimulation failed to improve the ICSI outcome.

## Introduction

Ovarian stimulation for assisted reproduction technology (ART) has three components: induction of multifollicular growth with gonadotropins, suppression of luteinizing hormone (LH) surge to prevent ovulation before egg retrieval and replacing the suppressed LH activity to induce oocyte maturation, which is also known as triggering. It is believed that the mode of triggering has a significant impact on the efficacy and safety of the ART treatment (1).

Human chorionic gonadotropin (hCG) is the traditional agent used to trigger oocyte maturation. Similarity between the beta subunits of LH and hCG molecules enable the latter to stimulate LH receptors on granulosa cells. However, the half-life of hCG is longer than LH and it induces longer stimulation of multiple corpora lutea following oocyte retrieval. This is associated with an increased risk of ovarian hyperstimulation syndrome (OHSS), a major risk of ovarian stimulation (2-6).

A single bolus of a gonadotropin releasing hormone agonist (GnRH-a) also induces an endogenous LH surge. The short duration of the GnRH-a induced LH surge leads to luteolysis and significantly decreases the risk of OHSS. However, luteolysis is associated with decreased pregnancy and increased miscarriage rates following fresh embryo transfer in GnRH-a triggered cycles (7-10).

The addition of a small dose of hCG for luteal phase support restores clinical outcome to some extent, but may increase the risk of OHSS, which justifies triggering without hCG in women at risk for OHSS (11).

Another suggested advantage of GnRH-a is the induction of an endogenous follicle stimulating hormone (FSH) surge simultaneous with the LH surge. The use of a GnRH-a to induce both endogenous LH and FSH surges, and hCG trigger simultaneously known as the “dual trigger”, has been suggested to improve ART outcomes (12-15).

It is suggested that addition of a GnRH agonist to the hCG trigger in women with low ovarian reserve could improve the *in vitro* fertilisation (IVF) outcome (16, 17). However, whether the dual trigger is beneficial over the traditional hCG trigger for the common ART patient is uncertain.

The present study aims to compare the laboratory and clinical outcomes of standard dose hCG trigger with a dual trigger of hCG and 1 mg leuprolide acetate.

## Materials and Methods

The Koc University Clinical Research Ethics Committee, Istanbul, Turkey approved the protocol of this retrospective cohort study (2019.269.IRB1.049). All the patients had signed an informed consent for study participation. 

Between January 2018 and September 2018, all women who planned to undergo egg retrieval for IVF/intracytoplasmic sperm injection (IVF/ICSI) at the Koc University Assisted Reproduction Centre, except for those at high risk for OHSS, were given the dual trigger in the context of another study on granulosa cell function. These patients constituted the dual trigger group. Women who received only the recombinant hCG (rhCG) trigger within three months immediately before and three months immediately after the dual trigger period constituted the hCG group. The authors were blinded to the pregnancy outcomes at the time of matching.

The dual trigger consisted of 1 mg leuprolide acetate (Lucrin Daily, Abbott, USA, equivalent to 0.1 mg Decapeptyle) and 250 mcg (6000 IU) rhCG (Ovitrelle, Merck, Germany), while the conventional trigger was 250 mcg (6000 IU) rhCG.

Women >45 years of age and with a history of recurrent pregnancy loss were excluded

Gonadotropins were started on the 2^nd^ or 3^rd^ day of the patient’s menstrual cycle after ruling out ovarian or endometrial pathology by a transvaginal ultrasound (TVUS) scan. The starting (rFSH) (Gonal F, Merck, Germany) dosage ranged between 225 and 300 IU/day, according to ovarian reserve and body weight. Ovarian response to gonadotropins was evaluated by TVUS and serum oestradiol levels on the 5^th^ or 6^th^ day of stimulation and every 1-3 days afterwards, based on clinical judgment. Daily administration of 25 mg GnRH antagonist (Cetrotide, Merck KGaA, Germany) was started when the leading follicle diameter reached 14 mm or serum oestradiol level exceeded 200 ng/ml. Final oocyte maturation was triggered when two leading follicles were >17 mm. Transvaginal egg retrieval was performed 36 hours after the trigger. Conventional ICSI was carried out and all embryos were cultured until the blastocyst stage. Luteal phase support with 90 mg vaginal micronized progesterone gel twice a day (Crinone 8%, Merck, Germany) was started on the evening of egg retrieval and continued until a negative pregnancy test or the 6th week of gestation.

Clinical pregnancy was defined as visualization of a gestational sac with a foetal heart beat by ultrasound at 6-7 weeks after embryo transfer. Ongoing pregnancy was defined as a pregnancy that proceeded beyond the 20^th^ gestational week. Oocyte maturation rate referred to the proportion of metaphase II (MII) oocytes to all collected oocytes per cycle. Implantation rate (IR) was calculated per cycle as the number of embryos with heart beat divided by the number of blastocysts transferred.

### Statistical analysis

Continuous variables were defined with mean (standard deviation) or median (25^th^-75^th^ percentile), and were compared between the groups with the t test or Mann-Whitney U test depending on distribution characteristics. Categorical variables were defined with numbers and percentages, and were compared between the groups with the chi-square test and its derivatives as appropriate. P<0.05 were considered statistically significant.

## Results

The study included 50 women in each group. Baseline characteristics of both groups were similar (Table 1). 

As shown in Table 2, the median number of oocytes collected (8 vs. 7, P=0.33), MII oocytes (6 vs. 5.5, P=0.41), blastocysts (1 in both groups), fertilisation (70% vs. 77%) and blastulation (30% vs. 28%) rates were similar in the dual trigger and hCG groups, respectively. 

Fresh embryo transfer was performed in 30 out of 50 (60%) women in the dual trigger group and in 26 out of 50 (52%) women in the hCG group (P=0.43). Clinical pregnancy rate (CPR, 28% vs. 22%, P=0.49) and ongoing pregnancy rate (OPR, 22% vs. 20% P=0.63) per woman were similar in the dual and hCG trigger groups, respectively. Pregnancy rate per transfer was 53.3% in the dual group and 53.8% in the hCG trigger group (P=0.96). CPR per transfer was 46.7% in the dual group and 42.3% in the hCG group (P=0.74). Both groups had a miscarriage rate of 8% and there were no cases of OHSS during the course of the study.

**Table 1:** Baseline characteristics of women in the dual trigger and hCG only groups


Baseline characteristics	Dual trigger (n=50)	hCG only (n=50)	P value

Age (Y)	33 (29-38)	33.5 (30-38)	0.90
Parity	0 (0-0)	0 (0-0)	0.18
Number of previous miscarriages	0 (0-0)	0 (0-0)	0.71
Number of previous failed IVF cycles	0 (0-1)	0 (0-1)	0.46
Duration of infertility (Y)	2 (1.5-4)	2.5 (2-4)	0.22
Cause of infertility			0.36
Ovulatory disorder	2	2	
Low ovarian reserve	14	10	
Tubal factor	2	2	
Endometriosis	3	3	
Male factor	13	8	
Unexplained	10	20	
Secondary infertility	6	5	
Body mass index (kg/m^2^)	23.7 (22.1-26.5)	23.1 (20.4-26-6)	0.34
Antral follicle count	11.5 (5.7-17.2)	8 (5-12)	0.13
Oestradiol level on trigger day (pg/ml)	1188 (678.8-1842.8)	1103 (780.8-1690)	0.61
Progesterone level on trigger day (ng/ml)	0.50 (0.25-0.63)	0.42 (0.29-0.70)	0.71
LH level at trigger day	2.6 (1.55-4.1)	3.5 (1.47-6.97)	0.34
Endometrial thickness (mm)	10 (7.9-11.8)	9.9 (8.5-11.9)	0.75
Gonadotropin starting dose (IU)	300 (281.2-300)	300 (300-300)	0.34
Total dose of gonadotropin	2329 (1950-2700)	2400 (2100-2944)	0.22
Duration of gonadotropin (days)	8 (7-10)	8 (8-10)	0.15


All values are median (25^th^-75^th^ percentile). hCG; Human chorionic gonadotropin, IVF; *In vitro* fertilisation, and LH; Luteinizing hormone

**Table 2:** Comparison of outcomes between the dual trigger and hCG only groups


Outcome	Dual trigger (n=50)	hCG only (n=50)	P value

Number of oocytes*	8 (4.75-8)	7 (4.75-10)	0.33
Number of MII oocytes*	6 (3-6)	5.5 (3-9)	0.41
Oocyte maturation rate*	0.80	0.75	0.90
Number of two pronuclear fertilised oocytes*	4 (2-4)	4 (2-7)	0.72
Fertilisation rate	0.70	0.77	0.47
Number of blastocysts*	1 (0-1)	1 (0-3)	0.77
Blastulation rate	0.30	0.28	0.33
Number of embryos transferred*	1 (0-1)	1 (0-1)	0.39
Number of frozen embryos*	1 (0-1)	0 (0-2)	0.92
Positive pregnancy test (pregnancy rate)	16/50 (32%)	14/50 (28%)	0.66
Pregnancy pate per transfer	16/30 (53.3%)	14/26 (53.8%)	0.96
CPR	14/50 (28%)	11/50 (22%)	0.49
CPR per transfer	14/30 (46.7%)	11/26 (42.3%)	0.74
IR	14/33 (42.4%)	11/28 (39.2%)	0.56
Number of miscarriages	4/50 (8%)	4/50 (8%)	0.64
LBR	11/50 (22%)	10/50 (20%)	0.80
LBR per transfer	11/30 (36.6%)	10/26 (38.4%)	0.42


*; Values are median (25^th^-75^th^ percentile), hCG; Human chorionic gonadotropin, CPR; Clinical pregnancy rate, MII; Metaphase II, IR; Implantation rate, and LBR; Live birth rate.

Fresh embryo transfer was performed in 30 out of 50 (60%) women in the dual trigger group and in 26 out of 50 (52%) women in the hCG group (P=0.43). Clinical pregnancy rate (CPR, 28% vs. 22%, P=0.49) and ongoing pregnancy rate (OPR, 22% vs. 20% P=0.63) per woman were similar in the dual and hCG trigger groups, respectively. Pregnancy rate per transfer was 53.3% in the dual group and 53.8% in the hCG trigger group (P=0.96). CPR per transfer was 46.7% in the dual group and 42.3% in the hCG group (P=0.74). Both groups had a miscarriage rate of 8% and there were no cases of OHSS during the course of the study.

## Discussion

In our study, universal use of dual trigger did not seem to provide any benefit regarding oocyte yield oocyte maturation, fertilisation, blastulation, implantation or CPR/ OPR compared to the hCG only trigger. However, the small number of samples is the shortcoming of this study. 

Effectiveness of dual triggering compared to hCG only or GnRH a only triggering has been investigated in a number of studies that vary greatly in design, methods and outcomes. Two randomised clinical trials (RCT) studied the effect of dual trigger in normo-responders. In the first one, Decleer et al. (18) studied 120 women <38 years of age who did not have polycystic ovarian syndrome or endometriosis. The mean number of retrieved oocytes were similar between the dual trigger (5000 IU hCG and 0.2 mg triptorelin acetate) and 5000 IU hCG trigger alone groups, respectively. The shortcoming of their study was the focus on day-3 embryos that had excellent quality. This subjective perception of excellence did not translate into better clinical outcomes as IR and OPR did not meet statistical significance between the dual trigger and hCG only groups. Moreover, day-3 embryo quality could be a poor predictor of blastulation, and the number of good quality day-3 embryos is a questionable outcome measure (19-21). 

Eftekhar et al. (22) randomized 192 normal responders to receive dual trigger or hCG only trigger. Although the mean number of oocytes (10.85 vs. 9.35) and embryos (6.86 vs. 5.34) were statistically higher in the dual trigger compared to the hCG only group, there were no significant differences between implantation or CPR between the dual trigger and hCG only groups, respectively. In another RCT, Kim et al. (23) compared dual trigger and hCG trigger alone for 60 women in each group. They observed that although the number of oocytes retrieved, fertilised oocytes and good quality embryos were similar in both groups, embryo IR (24.7% vs. 14.9%), CPR per cycle (53.3% vs. 33.3%) and live birth rate (LBR) (50.0% vs. 30.0%) were significantly higher in the dual trigger group compared to the hCG only group, respectively. They concluded that combined administration of GnRH a with rhCG might be beneficial in improving endometrial receptivity and pregnancy rates in GnRH antagonist cycles for IVF.

Ding et al. (24) conducted a systemic review and meta-analysis to investigate the efficacy of dual trigger compared to hCG alone. In their four eligible RCTs that included 527 women, they concluded that dual trigger was equivalent to hCG in triggering oocyte maturation and may be beneficial in improving reproductive outcomes; however, they emphasized that further intensive RCTs are needed to investigate the efficacy of dual trigger. 

Lin et al. (25) retrospectively compared the hCG only trigger and dual trigger in 376 normo-responder women, and reported that dual trigger significantly improved LBR. In another study, they evaluated the outcome of dual trigger in 427 cycles with fresh embryo transfer in patients with diminished ovarian reserve (antral follicle count of <5 or serum AMH level of <1.1 ng/ml) (17). They reported significantly higher fertilisation rate, clinical pregnancy and LBR with dual trigger compared to hCG only triggering.

Schachter et al. (26) examined the effect of dual trigger in a RCT of 200 cycles in women with history of at least one failed IVF/ICSI cycle on the GnRH-a long protocol. Although the mean number of oocytes (7.9 vs. 9.9) and embryos (4.7 vs. 5.7) were similar between the dual trigger (5000 IU hCG plus 0.2 mg Triptorelin) and control (5000 IU hCG) groups, there was a higher rate of OPR per transfer reported in the dual trigger group with marginal significance. 

Fabris et al. (27) studied 81 patients who had more than 50% immature oocytes in a previous rhCG only triggered ART cycle. The same women were given dual trigger in subsequent 81 cycles. Although they reported a significantly higher number of total and MII oocytes retrieved in the dual trigger group, it should be noted that any intervention almost always provides significant improvement in the second round of before-after studies where the first cycles are selected from those with particularly bad results. These findings are most likely explained by regression to the mean phenomenon, rather than a true biological effect (28, 29). Similarly, Griffin et al. (30) recruited 27 women with history of more than 25% immature oocytes (germinal vesicle or metaphase I) in their previous IVF cycles when triggered with hCG alone and compared the outcome of dual triggering with their previous cycle in a retrospective study. The proportion of mature oocytes retrieved was almost double with the dual trigger protocol compared to their previous hCG only trigger cycle (75% vs. 38.5%, OR: 2.51). However, similar to the Fabris et al. (27) study, the increase in oocyte maturation rate could likely be attributed to regression to the mean phenomenon.

Zhang et al. (31) compared dual trigger with hCG trigger only in a retrospective cohort study of 1350 poor responder patients diagnosed according to the Bologna criteria for poor responders. They reported increased numbers of mature oocytes with the dual trigger; however, fertilisation rate, number of viable embryos, implantation, and clinical pregnancy and miscarriage rates did not significantly differ between the groups. 

In summary, most studies reported improved intermediate outcomes rather than clinically relevant endpoints such as IR or OPR, whereas RCTs and our study reported similar clinical outcomes with dual and hCG only triggering. In addition, another RCT that assessed the isolated effect of FSH exposure on the day of ovulation trigger also failed to demonstrate a beneficial effect on OPR/LBR over hCG triggering alone (32).

n the present study, we used dual triggering for all women except those who were at high risk for OHSS on the trigger day, regardless of ovarian reserve or their previous IVF history. Moreover, the authors were blind to the cycle and clinical outcomes during matching of the controls. Thus, selection bias was reduced by avoiding patient selection or physician preference. Still, the retrospective nature and the size of the study are the weaknesses of this study. On the other hand, use of any hCG, alone or in combination with another agent, in patients at high risk for OHSS is not currently advised (33). Thus, this may not be a weakness but a choice that helps the study more aptly reflect clinical practice

## Conclusion

Based on our study and previous RCTs, universal use of dual triggering does not seem to result in improved oocyte yield, oocyte maturation, fertilisation, IR, and CPR or OPR. Studies on dual triggering show conflicting results on different patient groups; thus, its benefit for all women who undergo IVF/ICSI lacks robust evidence and large, well-designed trials should be conducted.
